# Supplementary material for: Auditory experience controls the maturation of song discrimination and sexual response in Drosophila
Source: eLife. 2018 Mar 20;7:e34348. doi: 10.7554/eLife.34348 (PMC5860867; doi:10.7554/eLife.34348)
Supplement: Supplementary file 1: — The genotypes used in figures are as follows. In Figures 5 and 6, the genotypes of females are listed, while the paired males are always wild type. [file elife-34348-supp1.docx]

**Supplementary file 1: Genotypes**

The genotypes used in figures are as follows. In Figures 5 and 6, the genotypes of females are listed, while the paired males are always wild type.

|  | **Animals** | **Genotype** |
| --- | --- | --- |
| Figure 1 | Wild type | *Canton S* |
| Figure 2 | Wild type | *Canton S* |
| Figure 3 | Wild type | *Canton S* |
| Figure 4 | Wild type | *Canton S* |
| Figure 5 | *Gad1* knockdown | *Gad1-GAL4/+; UAS-Gad1 RNAi/+* |
|  | Control | *Gad1-GAL4/+;+/+* |
| Figure 6 | *Rdl* knockdown | *tubP>GAL80>/+; NP2631-GAL4/UAS-Rdl-RNAi; dsx^FLP^/+* |
|  | Control | *tubP>GAL80>/+; NP2631-GAL4/+; dsx^FLP^/+* |
|  |  |  |
